# Contribution of *Staphylococcus aureus* Coagulases and Clumping Factor A to Abscess Formation in a Rabbit Model of Skin and Soft Tissue Infection

**DOI:** 10.1371/journal.pone.0158293

**Published:** 2016-06-23

**Authors:** Natalia Malachowa, Scott D. Kobayashi, Adeline R. Porter, Kevin R. Braughton, Dana P. Scott, Donald J. Gardner, Dominique M. Missiakas, Olaf Schneewind, Frank R. DeLeo

**Affiliations:** 1 Laboratory of Bacteriology, Rocky Mountain Laboratories, National Institute of Allergy and Infectious Diseases, National Institutes of Health, Hamilton, Montana, United States of America; 2 Rocky Mountain Veterinary Branch, Rocky Mountain Laboratories, National Institute of Allergy and Infectious Diseases, National Institutes of Health, Hamilton, Montana, United States of America; 3 Department of Microbiology, University of Chicago, Chicago, Illinois, United States of America; Rockefeller University, UNITED STATES

## Abstract

*Staphylococcus aureus* produces numerous factors that facilitate survival in the human host. *S*. *aureus* coagulase (Coa) and von Willebrand factor-binding protein (vWbp) are known to clot plasma through activation of prothrombin and conversion of fibrinogen to fibrin. In addition, *S*. *aureus* clumping factor A (ClfA) binds fibrinogen and contributes to platelet aggregation via a fibrinogen- or complement-dependent mechanism. Here, we evaluated the contribution of Coa, vWbp and ClfA to *S*. *aureus* pathogenesis in a rabbit model of skin and soft tissue infection. Compared to skin abscesses caused by the Newman wild-type strain, those caused by isogenic *coa*, *vwb*, or *clfA* deletion strains, or a strain deficient in *coa* and *vwb*, were significantly smaller following subcutaneous inoculation in rabbits. Unexpectedly, we found that fibrin deposition and abscess capsule formation appear to be independent of *S*. *aureus* coagulase activity in the rabbit infection model. Similarities notwithstanding, *S*. *aureus* strains deficient in *coa* and *vwb* elicited reduced levels of several proinflammatory molecules in human blood *in vitro*. Although a specific mechanism remains to be determined, we conclude that *S*. *aureus* Coa, vWbp and ClfA contribute to abscess formation in rabbits.

## Introduction

*Staphylococcus aureus* remains one of the most prominent human bacterial pathogens worldwide [1, 2]. These Gram-positive cocci cause a wide clinical spectrum of disease and/or syndromes, including endocarditis, bacteremia, pneumonia, toxic shock syndrome, osteomyelitis, and skin and soft tissue infections (SSTIs) [3–5]. The remarkable success of *S*. *aureus* as a human pathogen is facilitated by its vast arsenal of virulence factors and an ability to acquire antibiotic resistance readily [5, 6].

Coagulase (Coa) is one of the earliest described virulence factors of *S*. *aureus* [7], and is routinely used as a diagnostic tool to differentiate between two major species of *Staphylococcus*, i.e., coagulase-positive (*S*. *aureus*) and coagulase-negative (e.g., *S*. *epidermidis*) organisms. Relatively recently, a second *S*. *aureus* coagulase was discovered and named von Willebrand factor-binding protein (vWbp) [8]. Coa and vWbp display sequence and structure homology, particularly at the N-terminus [9]. Both proteins insert N-terminal residues into the prothrombin zymogen cleft, which triggers non-proteolytic activation by conformational transformation and formation of a staphylothrombin complex [10, 11]. The C-terminal domain (substrate recognition domain) of coagulase binds fibrinogen, which is transformed into fibrin and subsequently forms a fibrin clot.

Fibrin deposition is a process critical to abscess formation and thereby contributes to host defense against invading *S*. *aureus* [12]. The *S*. *aureus* coagulases have been linked previously to abscess development in murine systemic [13] and subcutaneous models of infection [14]. Clumping factor A (ClfA), although not a coagulase, is a fibrinogen binding protein that can promote fibrinogen-dependent platelet aggregation and adherence of *S*. *aureus* to fibrin [15, 16]. Similar to the coagulases, a role for ClfA in *S*. *aureus* abscess formation has been demonstrated in murine models of *S*. *aureus* virulence [17–19].

Rabbit models of *S*. *aureus* infection were used historically to investigate virulence and host-pathogen interactions, but were replaced largely by mouse infection models. Although neither mouse nor rabbit innate immune systems faithfully recapitulate that of humans, there are characteristics of the rabbit innate immune system—especially those of granulocytes—that seem more closely aligned with those of humans by comparison. A role for coagulases and ClfA has not been reported in a rabbit model of *S*. *aureus* SSTI. To that end, we evaluated the role of *S*. *aureus coa*, *vwb*, and *clfA* in a rabbit skin abscess model.

## Materials and Methods

### Ethics statement

All animal studies and procedures were approved by the Animal Care and Use Committee at Rocky Mountain Laboratories, National Institute of Allergy and Infectious Diseases (NIAID) under protocols 2011–92 and 2012–027, and conformed to the guidelines of the National Institutes of Health (NIH).

Human venous blood was obtained from healthy donors according to a protocol approved by the Institutional Review Board for Human Subjects, NIAID, NIH. Studies were conducted according to the policies provided in the Declaration of Helsinki, and each volunteer provided written informed consent prior to participation in the study.

### Bacterial strains and growth conditions

*S*. *aureus* Newman wild-type and isogenic *coa* (Δ*coa*), *vwb* (Δ*vwb*), and *coa*/*vwb* (Δ*coa*/Δ*vwb*) deletion strains, and a *clfA* transposon mutant strain (Δ*clfA*), were described previously [13, 20, 21]. Briefly, the pKOR1 allelic replacement system was used to create the *S*. *aureus* Δ*coa*, Δ*vwb* and Δ*coa*/Δ*vwb* deletion strains [13, 22], and the mariner-based *bursa aurealis* transposon system was used to construct the Δ*clfA* strain [21]. All *S*. *aureus* strains used for these studies have been phenotypically evaluated for their ability to clot whole blood [13]. We used *S*. *aureus* strains Newman, Δ*coa*, Δ*vwb*, and Δ*coa*/Δ*vwb* since they were readily available and used previously in murine models of *S*. *aureus* abscess formation [13, 21]. Bacteria were cultured in trypticase soy broth (TSB; Difco, Detroit, MI) at 37°C with constant shaking at 225 rpm. Overnight cultures were diluted 1:200 into fresh TSB and grown to early stationary (OD_600_ ~ 2.0) growth phase prior to use in assays.

### Rabbit skin and soft tissue infection model

Animal experiments were performed as described [23]. Briefly, bacteria were cultured to early stationary phase of growth and then pelleted by centrifugation. Cells were washed twice with Dulbecco’s phosphate-buffered saline (DPBS; Sigma-Aldrich, St. Louis, MO) and suspended in sterile DPBS at 5 × 10^9^ colony-forming units (CFU)/ml. The *S*. *aureus* dose used in this study was determined empirically in rabbits and results in reproducible abscesses that are easily evaluated by gross morphology [23]. *S*. *aureus* inocula were verified by enumeration of CFUs on trypticase soy agar plates. Five rabbits (NZW, strain Cr1c:KBL; Western Oregon Rabbit Company, Philomath, OR) were used for each group and each group was infected with a different *S*. *aureus* strain. Rabbits were anesthetized and subsequently inoculated with 100 μl of bacterial suspension into the left and right flank (5 rabbits for each strain and thus 10 abscesses per strain), and 100 μl of DPBS was injected into lower right flank for use as a sham infection control. Animals were monitored daily and allowed food and water *ad libitum*. *S*. *aureus* inflammatory lesions were measured daily for 14 days with a caliper as described previously [23]. Experiments were repeated twice using an additional set of two animals per strain to assess *S*. *aureus* abscess CFUs on day 2 post infection, and one animal per strain/day was used for histopathology analysis. Animals were humanely euthanized prior to tissue excision in accordance with protocol approved by the Institutional Animal Care and Use Committee.

### Histopathology analysis

Abscesses with margins of surrounding tissue were excised and fixed in 10% neutral-buffered formalin for at least 48 hours and processed as described [24]. Tissues sections were stained with hematoxylin-eosin, Masson’s trichrome stain for capsule or Mallory’s phosphotungstic acid-hematoxylin for fibrin visualization [25]. Images of tissue sections were captured using an Olympus model BX-51 microscope and Olympus cellSens Dimension 1.13 software (Olympus, Center Valley, PA).

### Quantitative analysis of molecules produced in human whole blood in response to *S*. *aureus*

Bacteria at mid-logarithmic growth phase were pelleted by centrifugation, washed twice with Dulbecco’s phosphate-buffered saline (DPBS; Gibco/Life Technologies, Grand Island, NY) and suspended in sterile RPMI 1640 medium buffered with 10 mM HEPES (RPMI/H; Invitrogen/Life Technologies, Grand Island, NY). Bacteria were added to heparinized human blood at a final concentration of 1 × 10^6^ CFU/ml. A 1-ml sample of blood culture was taken immediately to serve as a time zero control and the remaining samples were incubated for 2 h at 37°C with gentle rotation. The blood-bacteria mixture was centrifuged at 1300 × *g* for 10 min at 25°C to collect plasma for analysis of inflammation molecules. Samples were stored at -80°C until shipped for analysis (Multi-Analyte Profiling (MAP) technology platform (HumanMap^®^ v.2.0; Myriad RBM, Inc., Austin, TX). Data sets were analyzed using a one-way ANOVA and Tukey’s post-test. The complete results of the HumanMap analysis are provided in S1 Table.

### Statistical analysis

All statistical analyses were performed using GraphPad Prism version 6.0 (GraphPad Software Inc., San Diego, CA). Data for abscess size were evaluated with a one-way ANOVA and Dunnett’s post-test to correct for multiple comparisons.

## Results

**Fig 1:**
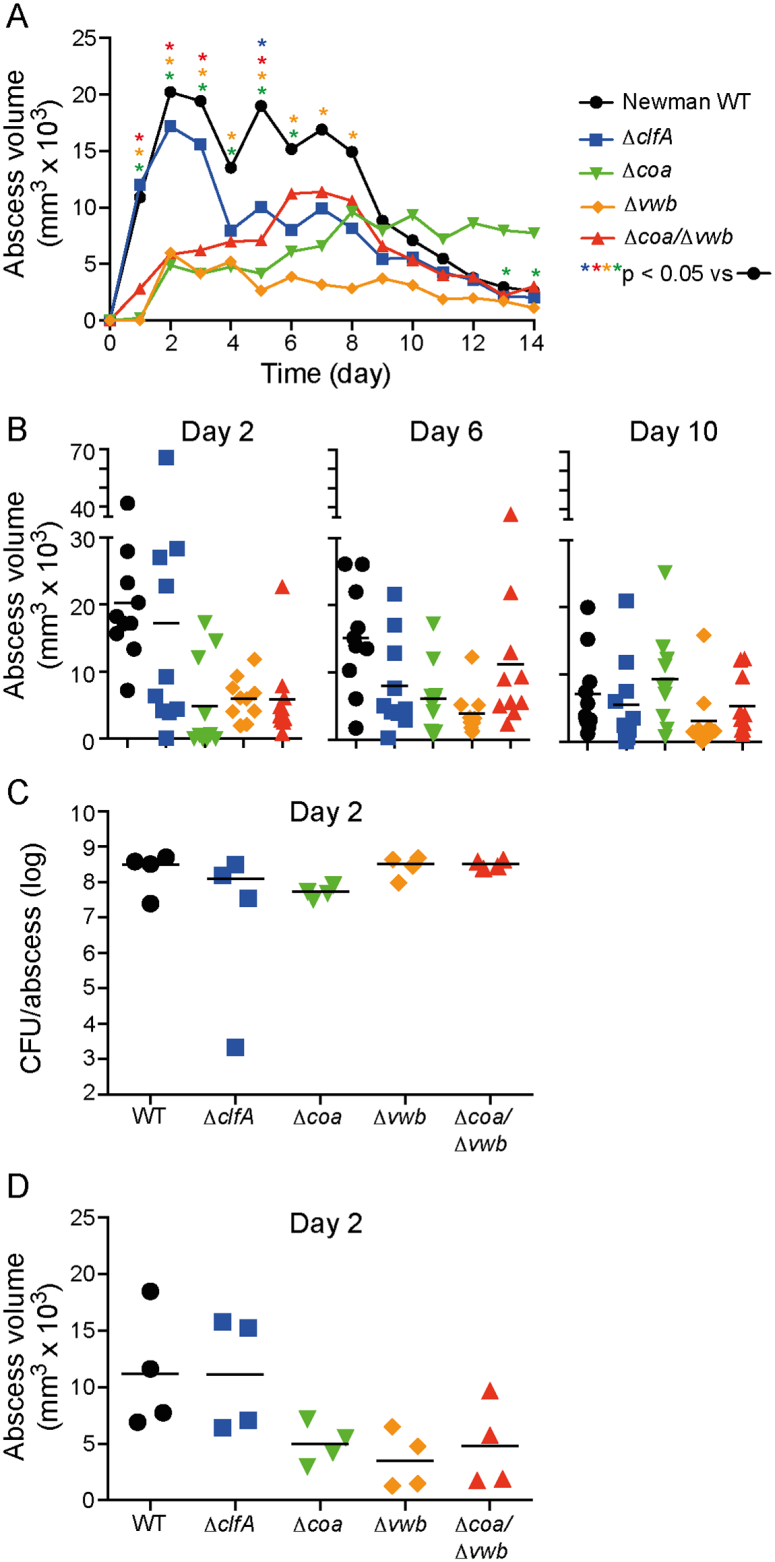
Contribution of *S*. *aureus coa*, *vwb* and *clfA* to formation of rabbit abscesses. (A) Average abscess volume for rabbits infected subcutaneously with *S*. *aureus* Newman wild-type (WT) or isogenic mutant strains as indicated. The volume of 10 abscesses per bacterial strain was measured daily following inoculation. (B) Individual abscesses plotted for selected days are depicted in panel A. (C) A separate set of 2 animals (4 abscesses/strain) was used to determine *S*. *aureus* CFU per abscess on day 2 post-infection and (D) the volume of rabbit abscesses. Each symbol represents a data point obtained from a single abscess. *P* values were calculated using a one-way ANOVA and Dunnett’s post-test.

SSTIs are among the most common manifestations of *S*. *aureus* disease. We previously developed a rabbit SSTI model to assess the relative contribution of USA300 virulence determinants to CA-MRSA pathogenesis [23]. Inasmuch as the *S*. *aureus* coagulases and ClfA contribute to fibrin deposition and are linked to abscess formation in murine infection models, we compared the ability of *S*. *aureus* Newman wild-type, Δ*coa*, Δ*vwb*, Δ*coa/*Δ*vwb*, and Δ*clfA* strains to cause abscesses in our rabbit SSTI model. Rabbits were infected by subcutaneous inoculation of *S*. *aureus* strains and abscess development (lesion size and assessment of gross morphology) was monitored daily for 14 days. All *S*. *aureus* strains tested caused formation of typical skin abscesses, as determined by gross morphology [23]. However, there were strain-dependent differences in abscess size (Fig 1A and 1B and S1 Fig). For example, abscesses caused by the Newman wild-type strain were significantly larger than those caused by Δ*coa*, Δ*vwb*, or Δ*coa/*Δ*vwb* strains on days 1, 2, 3, and 5 post-infection (*P* < 0.05, Fig 1A). Although abscesses caused by the Δ*clfA* strain were smaller than those caused by the wild-type strain (*P* < 0.05 on day 5), the difference was less pronounced compared to that of the coagulase negative strains (Fig 1A).

To determine if abscess size is associated with (or linked directly to) bacterial burden, we performed a second set of experiments to evaluate *S*. *aureus* CFUs in rabbit abscesses on day 2 following subcutaneous inoculation with each strain (Fig 1C and 1D). Unexpectedly, we found that CFUs per abscess were similar in all strains tested, indicating that the decreased abscess size (relative to wild-type) for the mutant strains was not due to a decrease in viability or more rapid bacterial clearance in this model. These findings contrast with those reported previously for Δ*clfA* [26, 27], Δ*vwb* and Δcoa strains [13, 28] in murine abscess models of *S*. *aureus* infection. It is possible differences in animal species (mouse versus rabbit) and infection models employed account for the differences in results with bacterial burden.

**Fig 2:**
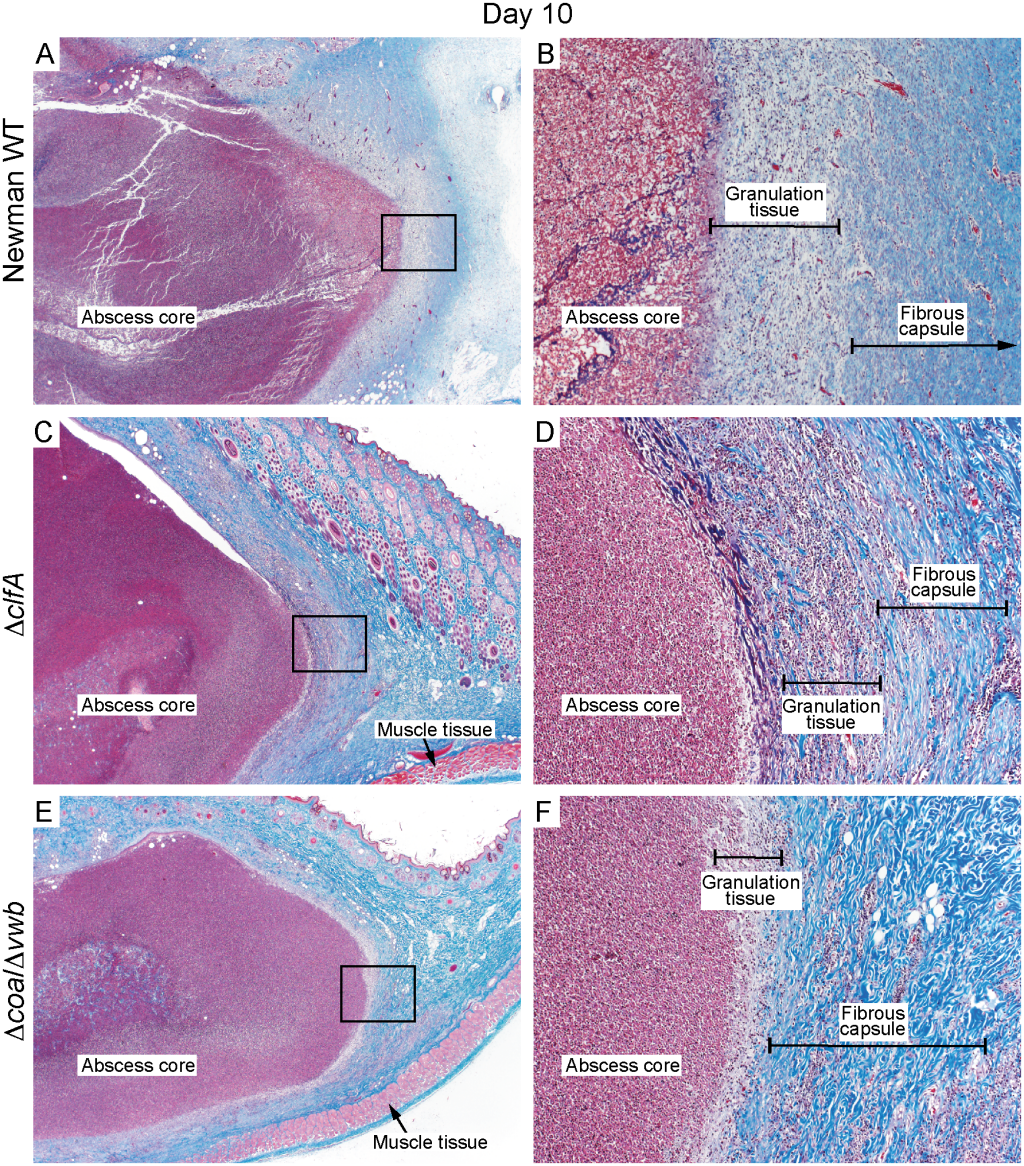
Histopathology of rabbit skin abscess caused by *S*. *aureus*. Histopathology sections represent skin abscesses caused by *S*. *aureus* Newman WT (A, B), Δ*clfA* (C, D) or Δ*coa/*Δ*vwb* (E, F) strains on day 10 post-infection. Abscess sections were stained with standard Masson’s trichrome stain to enhance fine structure detail of muscle tissues, collagen fibers and fibrin. (A, C and E) original magnification is 20×. (B, D, and F) 200× magnification of selected area (black rectangle) depicted in panels A, C or E.

**Fig 3:**
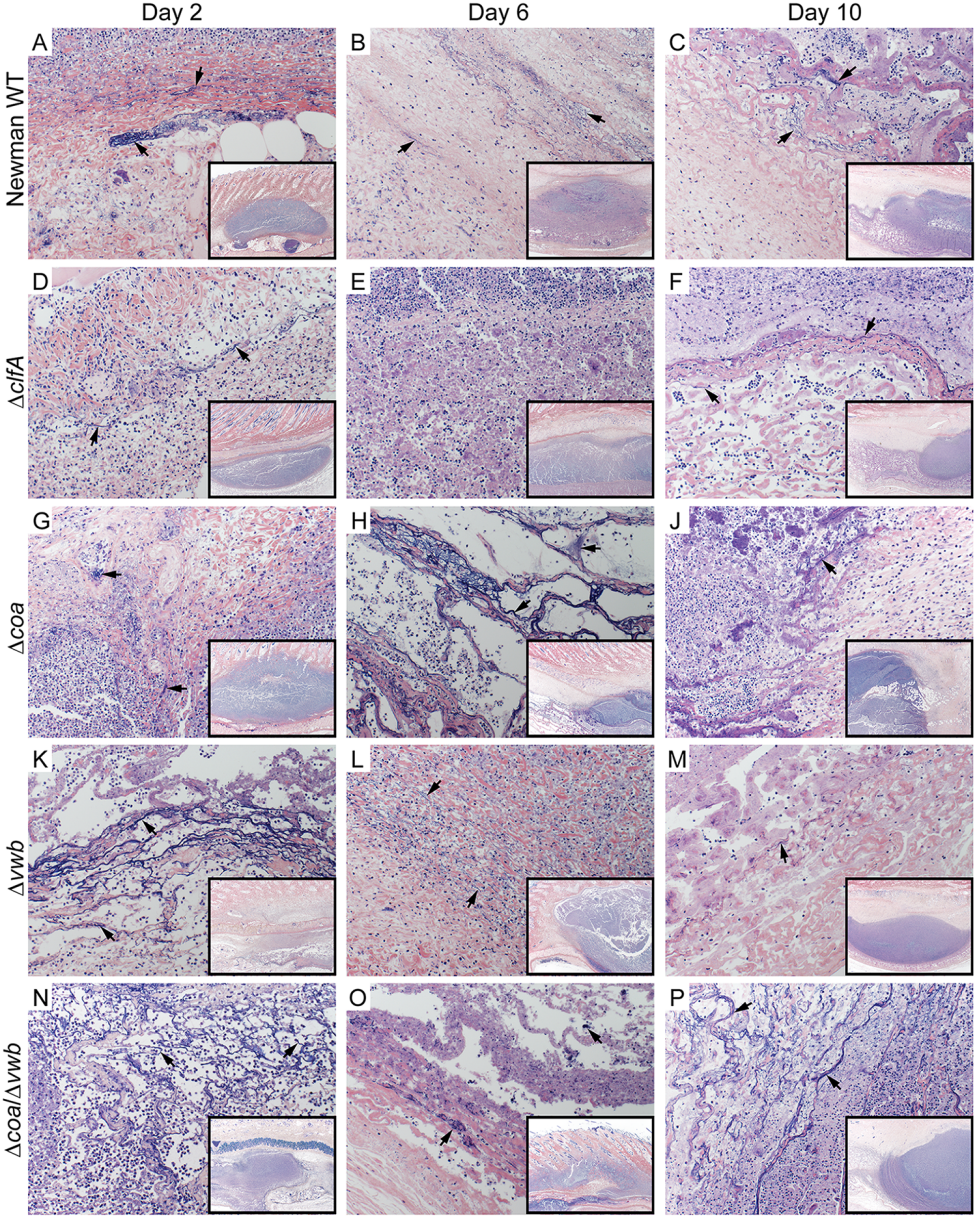
Fibrin deposition in rabbit skin abscess caused by *S*. *aureus* Newman. Representative sections of rabbit skin abscesses on Day 2 (A, D, G, K, N), Day 6 (B, E, H, L, O) and Day 10 (C, F, J, M, P) post infection. Abscesses from rabbits infected with *S*. *aureus* Newman WT (A-C), Δ*clfA* (D-F), Δ*coa* (G-J), Δ*vwb* (K-M) and Δ*coa/*Δ*vwb* (N-P). Tissue sections were stained with Mallory’s phosphotungstic acid-hematoxylin stain for visualization of fibrin (black arrows). Magnification is 200×. Inset image is the abscess at 20× (black rectangle).

Inasmuch as *S*. *aureus* coagulases and ClfA are involved in fibrin deposition, and since there were no apparent differences in bacterial numbers within abscesses, we next examined histological sections to ascertain differences in abscess fine structure (Fig 2 and Table 1). Abscesses were surgically excised on days 2, 6, and 10 following s.c. inoculation with *S*. *aureus*, and histopathology sections were processed and scored (Table 1). We found that all *S*. *aureus* strains tested caused formation of structurally discrete abscesses surrounded by a fully developed fibrous capsule by day 10, and that there were limited differences revealed by abscess histopathology—regardless of the *S*. *aureus* strain used for infection (Fig 2 and Table 1). Moreover, our data indicate that deposition of fibrin during abscess formation was mostly independent of Coa, vWbp and ClfA activity. Nonetheless, abscesses induced by Δ*clfA* had relatively weak fibrin deposition that was apparent only in abscesses excised on Day 2 and 10 (Fig 3). These data correspond with those in the mouse SSTI model, where fibrin deposition was apparent in abscesses induced by *S*. *aureus* when coagulase activity was blocked by dabigatran [14].

**Table 1:** Contribution of *coa*, *vwb* and *clfA* to structure and development of the abscess.

Strain	WT			Δ*clfA*			Δ*coa*			Δ*vwb*			Δ*coa/*Δ*vwb*			PBS ctrl	
Days post-infection	d2	d6	d10	d2	d6	d10	d2	d6	d10	d2	d6	d10	d2	d6	d10	d6	d10
Capsule granulation tissue	0/2	2/2	2/2	0/2	2/2	2/2	0/2	2/2	0/2	0/2	2/2	2/2	0/2	1/2	1/2	0/2	0/2
Fibrous capsule	0/2	0/2	2/2	0/2	2/2	2/2	0/2	0/2	2/2	0/2	0/2	2/2	0/2	0/2	2/2	0/2	0/2
Epithelialization	0/2	2/2	1/2	0/2	0/2	1/2	0/2	1/2	0/2	0/2	1/2	0/2	0/2	0/2	1/2	0/2	0/2
Vasculitis/vascular necrosis	2/2	2/2	2/2	1/2	0/2	0/2	2/2	2/2	0/2	2/2	2/2	1/2	2/2	2/2	0/2	0/2	0/2
Thrombosis	2/2	2/2	1/2	0/2	0/2	0/2	2/2	2/2	0/2	1/2	2/2	0/2	2/2	2/2	0/2	0/2	0/2
Coagulative necrosis	2/2	2/2	1/2	0/2	0/2	2/2	2/2	1/2	1/2	1/2	2/2	0/2	0/2	2/2	0/2	0/2	0/2
Extracellular bacteria	2/2	2/2	2/2	2/2	2/2	2/2	2/2	2/2	2/2	2/2	2/2	2/2	2/2	2/2	2/2	0/2	0/2
Intracellular bacteria	2/2	2/2	2/2	2/2	2/2	2/2	1/2	1/2	2/2	2/2	2/2	2/2	2/2	2/2	2/2	0/2	0/2
Compiled score	10/16	14/16	13/16	5/16	8/16	11/16	9/16	11/16	7/16	8/16	13/16	9/16	8/16	11/16	8/16	0/16	0/16

Abscesses were scored based on the presence (1) or absence (0) of a chosen feature based on 6 histopathology sections of two abscesses (3 each) for each bacterial strain. 0/2 = present in neither abscess; 1/2 = present in 1 of 2 abscesses; 2/2 = present in both abscesses.

**Fig 4:**
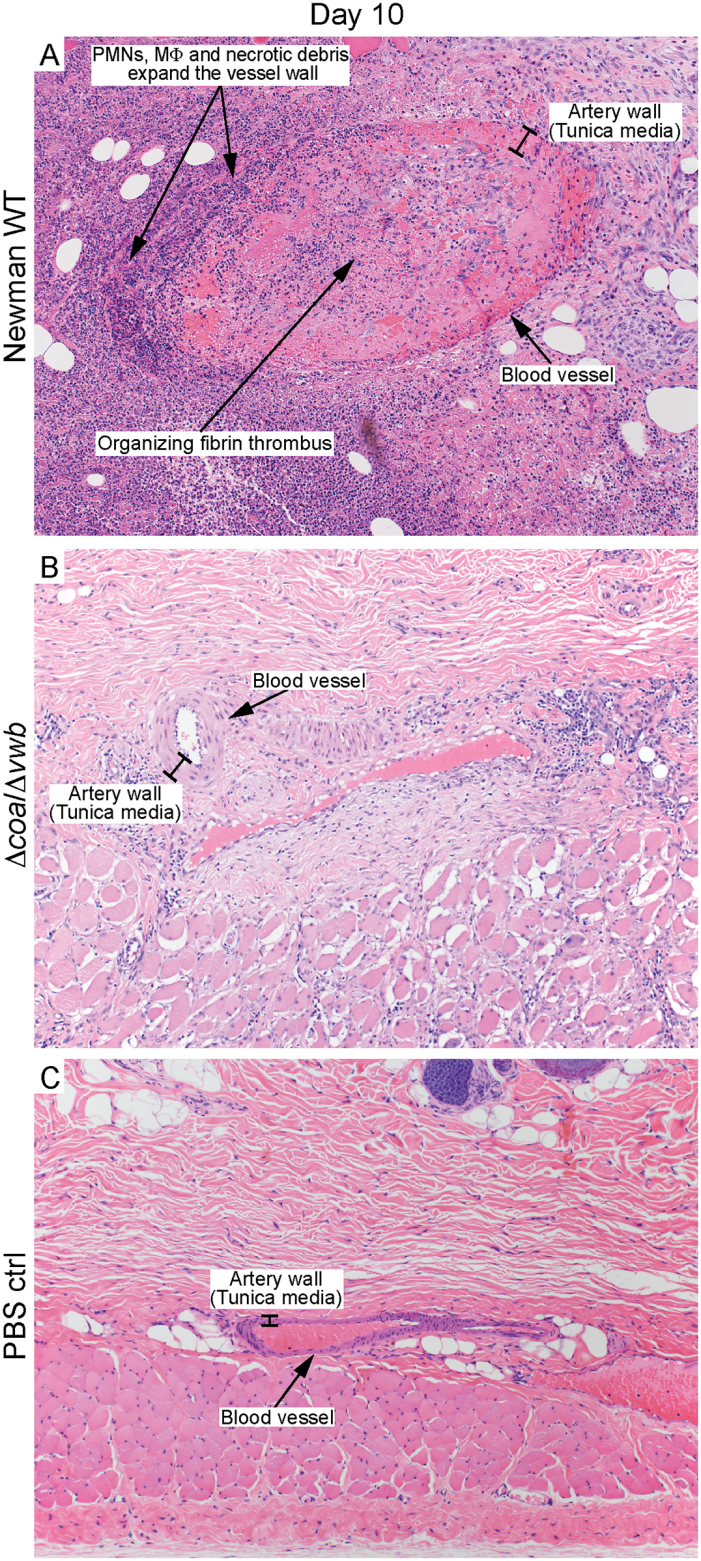
Vasculitis caused by *S*. *aureus* Newman WT. Histopathology sections of rabbit abscesses depicting vascular necrosis caused by *S*. *aureus* Newman WT (A), and an intact artery within a Δ*coa/*Δ*vwb* induced abscess (B) or PBS control (C) on day 10 post infection. Original magnification is 100×.

Abscesses caused by the Newman wild-type strain scored categorically highest on each day based on histopathology features, with few exceptions (Table 1). One notable distinction was that abscesses from rabbits infected with the Δ*clfA* strain had no evidence of thrombosis and weak vascular necrosis in the majority of sections analyzed. Although present at the earlier time points, vascular necrosis was also limited in tissue samples from abscess caused by coagulase deficient strains (Fig 4 and Table 1).

**Fig 5:**
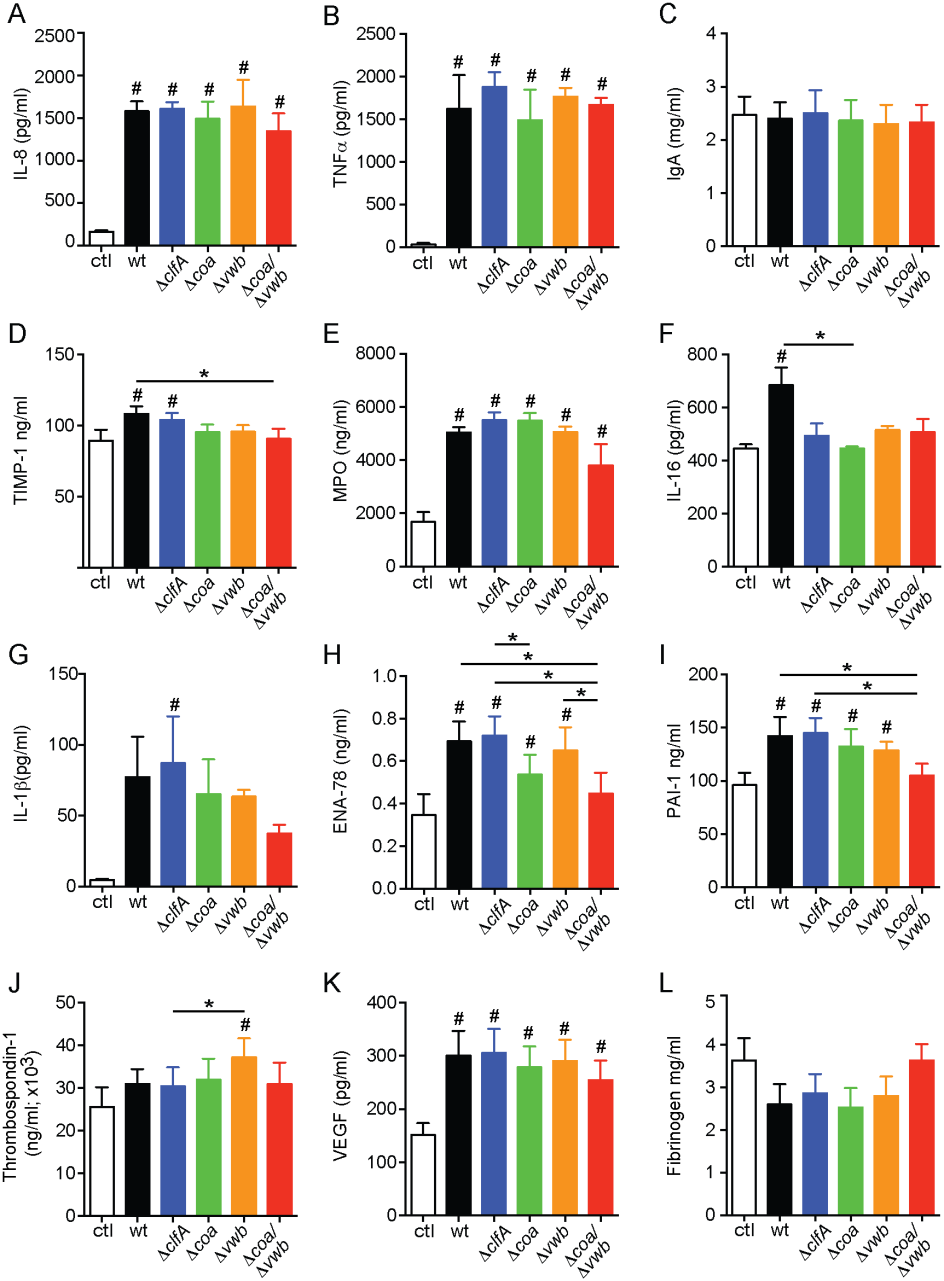
*S*. *aureus* Newman causes increased production of proinflammatory molecules in human whole blood. *S*. *aureus* was cultured in human heparinized blood for 2 h. Accumulation of proinflammatory molecules in plasma was evaluated by quantitative, multiplexed immunoassays (HumanMAP v2.0; Myriad RBM) as described in Materials and Methods section. Data represents average of 3 donors with one-way ANOVA and Tukey’s post-test used to determine statistical significance. *P < 0.05 for the selected pairs; # *p* < 0.05 compared to uninfected blood sample (ctrl).

The acute inflammatory response associated with *S*. *aureus* SSTI is triggered at least in part by production of pro-inflammatory signaling molecules and rapid recruitment of immune cells. To gain insight into the role of *S*. *aureus* coagulases and ClfA in the induction of host inflammation, we utilized a multi-analyte profiling approach to measure immune molecule production in human blood in response to *S*. *aureus* Newman wild-type, *ΔclfA*, *Δcoa*, *Δvwb*, and *Δcoa/Δvwb*. We performed the protein profiling experiments using human blood because there is paucity of reagents available to analyze a comprehensive panel of rabbit immune mediators. We also demonstrated previously that human and rabbit blood incubated with *S*. *aureus* have similar proinflammatory cytokine gene transcription profiles [29, 30]. As anticipated, several proinflammatory molecules were upregulated in blood samples incubated with *S*. *aureus* for 2 h compared to control blood lacking bacteria, including interleukin (IL)-8, myeloperoxidase (MPO), tumor necrosis factor (TNFα), and vascular endothelial growth factor (VEGF) (Fig 5 and S1 Table). There were also notable differences in levels of proinflammatory molecules elicited by *S*. *aureus* mutant and wild-type strains tested. For example, there was reduced expression of key proinflammatory mediators (IL-1β, MPO, PAI-1 and ENA-78) in human blood incubated with the *S*. *aureus Δcoa/Δvwb* strain compared to the wild-type Newman strain (Fig 5). If this phenomenon can be extended to host responses in tissues, it could provide an explanation in part for diminished pathology caused by the mutant strains in the rabbit SSTI model of infection, albeit this hypothesis requires further investigation.

## Discussion

The rabbit is historically the classical animal model for studying *S*. *aureus* pathogenesis [31, 32] and has been used to model a diversity of diseases and syndromes such as endocarditis, pneumonia, sepsis, and toxemia [33–37]. We recently developed a rabbit model of skin and soft tissue infection [23] to study the contribution of *S*. *aureus* leukotoxins to abscess formation. *In vitro* studies indicate that susceptibility of rabbit cells to several *S*. *aureus* secreted leukotoxins and hemolysins approximates that of human cells more closely than those of murine origin [38, 39]. For example, mouse leukocytes are less susceptible (7–10 fold) to the cytolytic effects of PVL compared with human or rabbit leukocytes, and purified PVL has been tested directly in a rabbit skin infection model [40].

Inasmuch as *S*. *aureus* Coa, vWbp and ClfA are linked previously to abscess formation following murine systemic infection, we employed the rabbit skin and soft tissue infection model to assess the role of coagulase in development of subcutaneous abscesses. Our data indicate that all three of these molecules contribute to the formation of *S*. *aureus* abscesses in the experimental rabbit infection model. However, we found that the direct contribution of *S*. *aureus* Coa and vWbp to capsule formation and fibrin deposition was limited (Figs 2 and 3) compared to that reported for the *S*. *aureus* murine kidney model [13]. There are a couple of potential explanations for differences observed between the infection models. First, there are significant differences in the host proinflammatory response to invading pathogens between mice and rabbits, and as an example, IL-8 is a factor critical for neutrophil recruitment in humans and rabbits but is absent in mice [41, 42]. While it is evident that proinflammatory mediators play a critical role in formation of *S*. *aureus* abscesses [43–46], it is unlikely that species-specific production of proinflammatory molecules contribute to the differences reported for the role of coagulases on abscess structure between the models. On the other hand, it is possible that the role of *S*. *aureus* coagulases on abscess structure differs depending on the anatomical location of the abscess, rather than the animal species tested. Renal abscesses form as a result of systemic infection, during which disseminated bacteria within host blood accumulate in blood filtration organs such as the kidney or liver. *S*. *aureus* commonly accumulates in the renal arcuate arteries and causes infarcts [47, 48]. The combination of bacteria and tissue damage elicits neutrophil and other immune cell infiltration, and facilitates formation of a mature abscess. By contrast, invading *S*. *aureus* are recognized early during SSTI by local keratinocytes and resident skin monocytes, which initiate cytokine signaling to promote immune cell recruitment [49]. This triggers influx of neutrophils to the infection site to initiate the process of abscess formation [12, 50]. The influx of neutrophils also contributes to increased vascular permeability at the site of inflammation [51, 52]. Since coagulases and clumping factor A function primarily through binding or modifying fibrinogen—one of the most abundant plasma glycoproteins [53]—it is possible that limited access to fibrinogen in subcutaneous tissue reduces the role of coagulases and/or ClfA in formation of the SSTI abscess compared to the kidney. Indeed, consistent with our findings in rabbits, a *S*. *aureus* strain deficient for *coa* and *vwb* formed smaller subcutaneous abscesses in murine SSTI, and inhibition of the staphylothrombin complex by dabigatran treatment did not prevent deposition of fibrin and fibrinogen within the *S*. *aureus* wild-type abscess capsule [14]. However, in that study, abscess structure was not assessed directly by histopathology following infection with the *S*. *aureus Δcoa/Δvwb* deletion strain. Nevertheless, more work is needed to determine if there are variations in organ-specific immune response and/or bacterial response that may influence abscess development. Collectively, the data obtained from our rabbit infection model confirm previous findings that Coa, vWbp and ClfA are involved in the pathogenesis of *S*. *aureus* SSTI and contribute to the host proinflammatory response to infection.

## Supporting Information

S1 Fig Rabbit abscess volume following infection with *S*. *aureus* wild type and Δ*coa*, Δ*vwb*, Δ*clfA*, and Δ*coa*/Δ*vwb* isogenic deletion strains. Scatter plot of abscess volumes from data shown in Fig 1A. Rabbits were infected subcutaneously with *S*. *aureus* Newman wild-type (WT) or isogenic mutant strains. The volume of 10 abscesses per bacterial strain was measured for 14 days following inoculation. Each symbol represents a data point obtained from a single abscess.
(TIF)

S1 Table Production of proinflammatory molecules in human whole blood after incubation with *S*. *aureus* Newman strain and its isogenic mutants. *S*. *aureus* was cultured in human heparinized blood up to 2 h. Accumulation of proinflammatory molecules in plasma was evaluated by quantitative, multiplexed immunoassays (HumanMAP v2.0; Myriad RBM) as described in Materials and Methods section. Data represents average of 3 donors ±SEM.
(DOCX)
