# Supplementary figures and images for: Contribution of Staphylococcus aureus Coagulases and Clumping Factor A to Abscess Formation in a Rabbit Model of Skin and Soft Tissue Infection
Source: PLoS One. 2016 Jun 23;11(6):e0158293. doi: 10.1371/journal.pone.0158293 (PMC4918888; doi:10.1371/journal.pone.0158293)

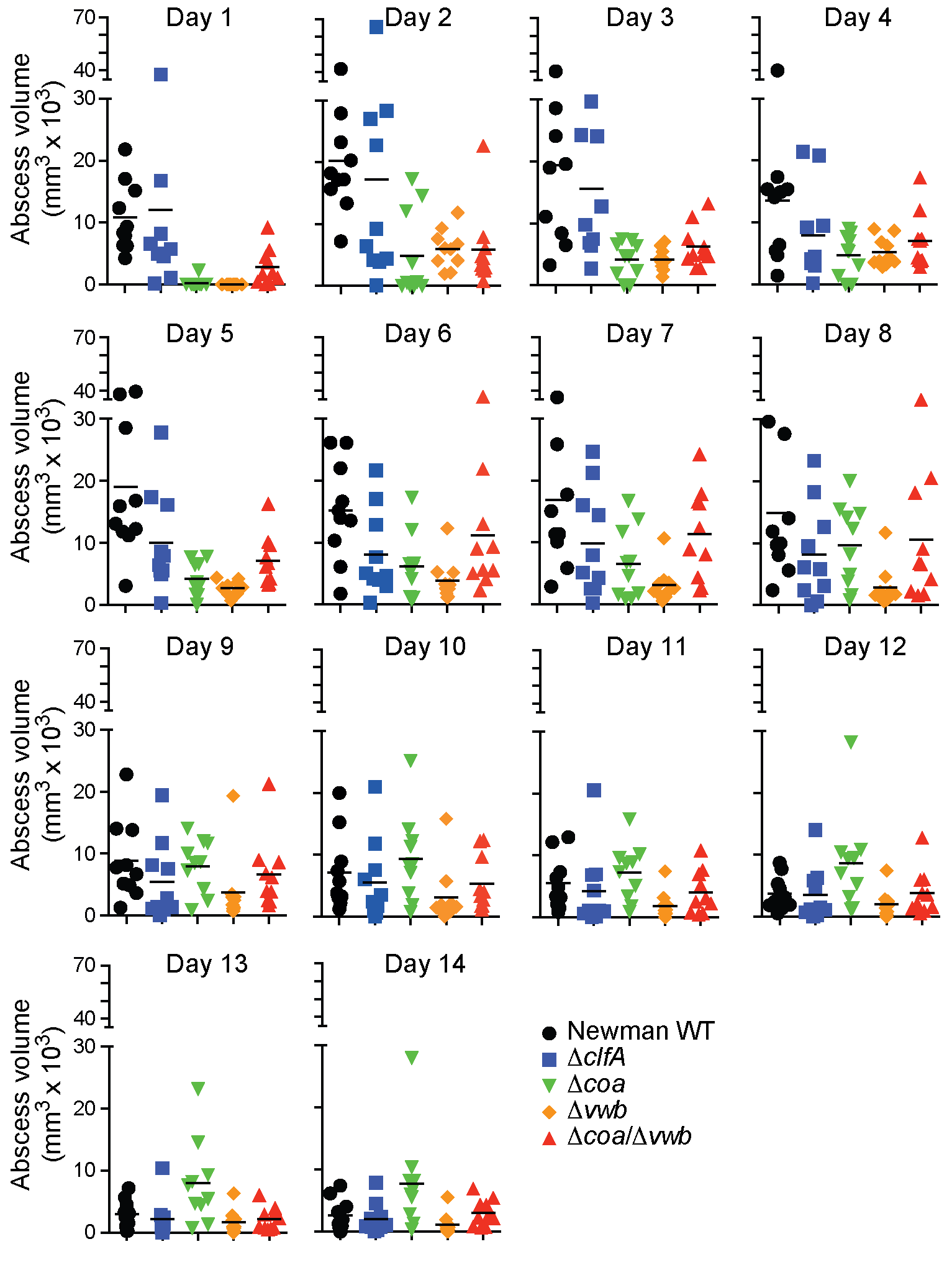

Supplement: S1 Fig — Scatter plot of abscess volumes from data shown in Fig 1A. Rabbits were infected subcutaneously with S. aureus Newman wild-type (WT) or isogenic mutant strains. The volume of 10 abscesses per bacterial strain was measured for 14 days following inoculation. Each symbol represents a data point obtained from a single abscess. (TIF) [file pone.0158293.s001.tif]
