# Supplementary material for: Contribution of Staphylococcus aureus Coagulases and Clumping Factor A to Abscess Formation in a Rabbit Model of Skin and Soft Tissue Infection
Source: PLoS One. 2016 Jun 23;11(6):e0158293. doi: 10.1371/journal.pone.0158293 (PMC4918888; doi:10.1371/journal.pone.0158293)
Supplement: S1 Table — S. aureus was cultured in human heparinized blood up to 2 h. Accumulation of proinflammatory molecules in plasma was evaluated by quantitative, multiplexed immunoassays (HumanMAP v2.0; Myriad RBM) as described in Materials and Methods section. Data represents average of 3 donors ±SEM. (DOCX) [file pone.0158293.s002.docx]

**S1 Table. Production of proinflammatory molecules in human whole blood after incubation with *S. aureus* Newman strain and its isogenic mutants.**

| **Analytes (units)** | **Average value (±SEM)** | | | | | | | |
| --- | --- | --- | --- | --- | --- | --- | --- | --- |
|  | **0 min** | | **2 h** | | | | | |
|  | **Ctrl** | **Newman WT** | **Ctrl** | **Newman WT** | ***ΔclfA*** | ***Δcoa*** | ***Δvwb*** | ***Δcoa/Δvwb*** |
| Adiponectin (μg/ml) | 3.13 (±0.61) | 2.97 (±0.55) | 3.07 (±0.59) | 3.00 (±0.60) | 3.13 (±0.63) | 2.87 (±0.64) | 2.83 (±0.58) | 3.03 (±0.68) |
| α1-Antitrypsin (AAT; mg/ml) | 1.33 (±0.32) | 1.35 (±0.27) | 1.36 (±0.39) | 1.25 (±0.30) | 1.36 (±0.30) | 1.30 (±0.29) | 1.31 (±0.28) | 1.41 (±0.26) |
| α2-Macroglobulin (A2Macro; mg/ml) | 1.04 (±0.06) | 1.07 (±0.07) | 1.06 (±0.04) | 1.03 (±0.04) | 1.06 (±0.07) | 1.09 (±0.06) | 1.02 (±0.04) | 1.03 (±0.03) |
| α-Fetoprotein (AFP; ng/ml) | <0.56* | <0.56* | <0.56* | <0.56* | 0.57 (±0.30) | <0.56* | <0.56* | <0.56* |
| Apolipoprotein(a) (Lp(a); μg/ml) | 32.67 (±11.5) | 34.67 (±10.8) | 31.33 (±9.6) | 31.00 (±10.3) | 33.33 (±10.3) | 28.67 (±8.57) | 29.00 (±10.0) | 28.33 (±7.88) |
| Apolipoprotein A-I (Apo A-I; mg/ml) | 1.18 (±0.17) | 1.21 (±0.24) | 1.18 (±0.21) | 1.23 (±0.19) | 1.21 (±0.17) | 1.10 (±0.16) | 1.14 (±0.18) | 1.23 (±0.19) |
| Apolipoprotein A-II (Apo A-II; ng/ml) | 190.00 (±14.2) | 192.00 (±27.1) | 205.67 (±23.6) | 207.00 (±24.5) | 184.00 (±10.4) | 166.00 (±19.4) | 182.67 (±20.1) | 196.67 (±20.9) |
| Apolipoprotein C-I (Apo C-I; ng/ml) | 180.00 (±15.1) | 196.00 (±25.7) | 191.00 (±20.5) | 189.00 (±22.5) | 193.00 (±20.7) | 171.00 (±22.9) | 184.67 (±22.6) | 192.67 (±22.9) |
| Apolipoprotein C-III (Apo C-III; μg/ml) | 147.00 (±14.5) | 152.00 (±25.7) | 148.00 (±22.4) | 154.67 (±29.9) | 142.00 (±14.5) | 134.33 (±25.3) | 146.67 (±25.5) | 153.00 (±20.7) |
| Apolipoprotein H (Apo H; μg/ml) | 250.67 (±20.7) | 240.00 (±9.29) | 245.33 (±9.39) | 254.33 (±24.0) | 237.00 (±19.3) | 225.67 (±24.4) | 239.33 (±17.8) | 253.33 (±19.2) |
| β2-Microglobulin (B2M; μg/ml) | 1.53 (±0.33) | 1.57 (±0.33) | 1.57 (±0.33) | 1.63 (±0.33) | 1.67 (±0.88) | 1.57 (±0.88) | 1.60 (±0.10) | 1.70 (±0.10) |
| Brain-Derived Neurotrophic Factor (BDNF; ng/ml) | 3.67 (±0.68) | 3.63 (±0.33) | 9.47 (±1.53) | 13.00 (±0.58) | 13.33 (±0.88) | 12.33 (±0.88) | 13.00 (±1.00) | 10.77 (±1.36) |
| C-Reactive Protein (CRP; μg/ml) | 0.57 (±0.26) | 0.62 (±0.29) | 0.56 (±0.25) | 0.58 (±0.25) | 0.62 (±0.28) | 0.59 (±0.27) | 0.66 (±0.31) | 0.66 (±0.33) |
| Cancer Antigen 125 (CA-125; U/ml) | <8.7* | <8.7* | <8.7* | <8.7* | <8.7* | <8.7* | <8.7* | <8.7* |
| Cancer Antigen 19-9 (CA-19-9; U/ml) | 8.27 (±2.25) | 8.70 (±0.60) | 7.80 (±0.79) | 7.73 (±1.13) | 6.87 (±1.11) | 9.57 (±0.76) | 7.47 (±1.23) | 9.30 (±1.56) |
| Carcinoembryonic Antigen (CEA; ng/ml) | 0.90 (±0.49) | 0.83 (±0.44) | 0.79 (±0.44) | 1.19 (±0.39) | 1.22 (±0.39) | 1.46 (±0.32) | 0.96 (±0.55) | 1.22 (±0.27) |
| CD 40 antigen (CD40; ng/ml) | 0.51 (±0.02) | 0.53 (±0.04) | 0.72 (±0.06) | 0.79 (±0.04) | 0.82 (±0.02) | 0.79 (±0.08) | 0.79 (±0.06) | 0.75 (±0.08) |
| CD40 Ligand (CD40-L; ng/ml) | 0.09 (±0.04) | 0.14 (±0.04) | 1.01 (±0.16) | 1.57 (±0.07) | 1.70 (±0.12) | 1.43 (±0.15) | 1.53 (±0.12) | 1.21 (±0.26) |
| Complement C3 (C3; mg/ml) | 0.79 (±0.07) | 0.76 (±0.07) | 0.76 (±0.05) | 0.74 (±0.06) | 0.79 (±0.06) | 0.75 (±0.05) | 0.74 (±0.05) | 0.80 (±0.03) |
| Creatine Kinase-MB (CK-MB; ng/ml) | 1.55 (±0.60) | 1.64 (±0.69) | 1.01 (±0.48) | 1.56 (±0.65) | 1.13 (±0.53) | 1.22 (±0.51) | 1.38 (±0.72) | 1.25 (±0.66) |
| EN-RAGE (ng/ml) | 21.33 (±7.69) | 15.67 (±2.40) | 38.33 (±12.2) | 88.33 (±26.0) | 48.00 (±5.69) | 50.33 (±18.3) | 46.00 (±15.0) | 59.67 (±24.6) |
| Eotaxin-1 (pg/ml) | 736.00 (±206) | 743.33 (±197) | 851.00 (±245) | 866.00 (±245) | 874.33 (±239) | 850.67  (±210) | 858.33 (±231) | 824.67 (±223) |
| Epithelial-Derived Neutrophil-Activating Protein 78 (ENA-78; ng/ml) | 0.57 (±0.18) | 0.61 (±0.20) | 0.35 (±0.10) | 0.69 (±0.09) | 0.72 (±0.09) | 0.54 (±0.09) | 0.65 (±0.11) | 0.45 (±0.10) |
| Erythropoietin (EPO; mIU/ml) | <17* | <17* | <17* | <17* | <17* | <17* | <17* | <17* |
| Factor VII (ng/ml) | 288.33 (±60.6) | 285.33 (±56.3) | 286.67 (±60.7) | 290.67 (±68.7) | 295.33 (±63.6) | 280.33 (±55.7) | 288.00 (±58.4) | 281.33 (±57.4) |
| Ferritin (FRTN; ng/ml) | 73.33 (±31.2) | 72.00 (±31.0) | 75.00 (±29.5) | 90.33 (±37.3) | 84.33 (±34.5) | 83.00 (±32.9) | 78.33 (±29.2) | 74.67 (±29.0) |
| Fibrinogen (mg/ml) | 3.47 (±0.52) | 3.57 (±0.52) | 3.63 (±0.52) | 2.60 (±0.47) | 2.87 (±0.45) | 2.53 (±0.45) | 2.80 (±0.45) | 3.63 (±0.38) |
| Glucagon-like Peptide 1, active (GLP-1 active; pg/ml) | <24* | <24* | <24* | <24* | <24* | <24* | <24* | <24* |
| Granulocyte Colony-Stimulating Factor (G-CSF; pg/ml) | <48* | <48* | <48* | <48* | <48* | <48* | <48* | <48* |
| Granulocyte-Macrophage Colony-Stimulating Factor (GM-CSF; pg/ml) | <21* | <21* | <21* | <21* | <21* | <21* | <21* | <21* |
| Growth Hormone (GH; ng/ml) | 0.99 (±0.61) | 0.96 (±0.61) | 0.98 (±0.61) | 1.00 (±0.61) | 1.01 (±0.64) | 0.97 (±0.58) | 1.02 (±0.64) | 0.92 (±0.55) |
| Haptoglobin (mg/ml) | 0.42 (±0.13) | 0.45 (±0.11) | 0.43 (±0.13) | 0.38 (±0.15) | 0.43 (±0.13) | 0.39 (±0.14) | 0.39 (±0.14) | 0.43 (±0.12) |
| Human Chorionic Gonadotropin beta (hCG; mIU/ml) | <1.1* | <1.1* | <1.1* | <1.1* | <1.1* | <1.1* | <1.1* | <1.1* |
| Immunoglobulin A (IgA; mg/ml) | 2.37 (±0.29) | 2.43 (±0.37) | 2.47 (±0.35) | 2.40 (±0.31) | 2.50 (±0.44) | 2.37 (±0.38) | 2.30 (±0.36) | 2.33 (±0.33) |
| Immunoglobulin E (IgE; U/ml) | 12.67^#^ | 10.33^#^ | 15.67^#^ | 12.67^#^ | 13.33^#^ | 9.33^#^ | 13.33^#^ | 14.3^#^ |
| Immunoglobulin M (IgM; mg/ml) | 2.07 (±0.77) | 1.96 (±0.72) | 2.03 (±0.78) | 1.92 (±0.68) | 1.89 (±0.71) | 1.93 (±0.76) | 1.77 (±0.69) | 1.99 (±0.71) |
| Insulin (μIU/ml) | 3.67 (±0.66) | 3.47 (±0.52) | 3.13 (±0.54) | 3.63 (±0.58) | 3.10 (±0.46) | 3.37 (±0.38) | 3.17 (±0.35) | 3.27 (±0.55) |
| Intercellular Adhesion Molecule 1 (ICAM-1; ng/ml) | 71.67 (±4.67) | 73.00 (±6.43) | 77.33 (±3.71) | 76.33 (±1.45) | 84.33 (±8.09) | 76.33 (±5.46) | 81.33 (±6.84) | 75.33 (±5.67) |
| Interferon γ (IFN-γ; pg/ml) | <2.5* | <2.5* | <2.5* | <2.5* | <2.5* | <2.5* | <2.5* | <2.5* |
| Interleukin-1α (IL-1α; ng/ml) | <0.002* | <0.002* | <0.002* | <0.002* | <0.002* | <0.002* | <0.002* | <0.002* |
| Interleukin-1β (IL-1β; pg/ml) | 4.27 (±0.35) | 4.50 (±0.32) | 4.70 (±0.74) | 77.33 (±28.39) | 87.00 (±33.06) | 65.33 (±24.31) | 63.33 (±4.67) | 37.33 (±6.06) |
| Interleukin-1 receptor antagonist (IL-1ra; pg/ml) | 1011.33 (±74.3) | 968.67 (±151) | 1155.67 (±125) | 1295.67 (±189) | 1370.00 (±121) | 1316.67 (±141) | 1336.67 (±126) | 1155.67 (±165) |
| Interleukin-2 (IL-2; pg/ml) | <41* | <41* | <41* | <41* | <41* | <41* | <41* | <41* |
| Interleukin-3 (IL-3; ng/ml) | <0.0051* | <0.0051* | <0.0051* | <0.0051* | <0.0051* | <0.0051* | <0.0051* | <0.0051* |
| Interleukin-4 (IL-4; pg/ml) | <35* | <35* | <35* | <35* | <35* | <35* | <35* | <35* |
| Interleukin-5 (IL-5; pg/ml) | <4.7* | <4.7* | <4.7* | <4.7* | <4.7* | <4.7* | <4.7* | <4.7* |
| Interleukin-6 (IL-6; pg/ml) | <4.5* | <4.5* | 1.90*(±1.90) | 60.67 (±21.1) | 67.00 (±23.3) | 58.67 (±20.3) | 93.67 (±30.1) | 55.67 (±14.2) |
| Interleukin-7 (IL-7; pg/ml) | <32* | <32* | <32* | 28.7 (±14.4) | 46.67 (±5.24) | 41.00 (±3.61) | 44.00 (±4.36) | 24.7 (±12.5) |
| Interleukin-8 (IL-8; pg/ml) | 3.00 (±1.50) | 3.97 (±0.65) | 165.67 (±15.4) | 1580.00 (±118) | 1606.67 (±81.9) | 1490.00 (±205) | 1633.33 (±316) | 1345.33 (±210) |
| Interleukin-10 (IL-10; pg/ml) | <6.8* | <6.8* | <6.8* | <6.8* | <6.8* | <6.8* | <6.8* | <6.8* |
| Interleukin-12 Subunit p40 (IL-12p40; ng/ml) | 0.40 (±0.03) | 0.400 (±0.03) | 0.440 (±0.07) | 0.460 (±0.07) | 0.460 (±0.07) | 0.480 (±0.04) | 0.460 (±0.04) | 0.430 (±0.04) |
| Interleukin-12 Subunit p70 (IL-12p70; pg/ml) | <44* | <44* | <44* | <44* | <44* | <44* | <44* | <44* |
| Interleukin-13 (IL-13; pg/ml) | <15* | <15* | <15* | <15* | <15* | <15* | <15* | <15* |
| Interleukin-15 (IL-15; ng/ml) | 0.81 (±0.0) | 0.75 (±0.03) | 0.88 (±0.05) | 0.78 (±0.08) | 0.93 (±0.09) | 0.86 (±0.03) | 0.81 (±0.0) | 0.80 (±0.01) |
| Interleukin-16 (IL-16; pg/ml) | 474.67 (±43.7) | 462.677 (±45.2) | 446.007 (±15.9) | 684.337 (±65.8) | 494.007 (±45.8) | 445.677 (±7.69) | 515.677 (±15.5) | 507.007 (±50.0) |
| Interleukin-17 (IL-17; pg/ml) | <3.1* | <3.1* | <3.1* | <3.1* | <3.1* | <3.1* | <3.1* | <3.1* |
| Interleukin-18 (IL-18; pg/ml) | 184.337 (±47.1) | 183.337 (±35.6) | 180.007 (±37.0) | 213.337 (±47.1) | 220.007 (±51.1) | 220.337 (±50.1) | 205.007 (±38.9) | 216.337 (±54.3) |
| Interleukin-23 (IL-23; ng/ml) | 1.43 (±0.72) | 1.13 (±0.57) | 0.73 (±0.73) | 1.53 (±0.77) | 1.90 (±0.20) | 1.90 (±0.20) | 1.33 (±0.67) | 1.63 (±0.92) |
| Leptin (ng/ml) | 9.94 (±3.99) | 9.93 (±4.01) | 8.67 (±3.21) | 8.57 (±3.24) | 8.47 (±3.23) | 8.63 (±3.21) | 8.93 (±3.46) | 8.47 (±3.30) |
| Macrophage-Derived Chemokine (MDC; pg/ml) | 402.00 (±123) | 387.00 (±109) | 461.67 (±140) | 482.67 (±90.7) | 513.33 (±121) | 469.67 (±87.1) | 505.33 (±121) | 460.67 (±135) |
| Macrophage Inflammatory Protein-1α (MIP-1α; pg/ml) | <29* | <29* | 182.67 (±35.0) | 2234.33 (±795) | 2563.33 (±767) | 2144.67 (±965) | 2686.67 (±696) | 1826.67 (±487) |
| Macrophage Inflammatory Protein-1β (MIP-1β; pg/ml) | 138.33 (±67.8) | 141.67 (±68.2) | 2956.67 (±800) | 11796.67 (±2573) | 12093.33 (±1971) | 10623.33 (±2942) | 15000.00 (±2730) | 11763.33 (±2519) |
| Matrix Metalloproteinase-2 (MMP-2; ng/ml) | 1683.33 (±130) | 1733.33 (±70.6) | 1656.67 (±95.6) | 1606.67 (±163) | 1646.67 (±132) | 1646.67 (±113) | 1653.33 (±110) | 1680.00 (±136) |
| Matrix Metalloproteinase-3 (MMP-3; ng/ml) | 14.33 (±2.73) | 14.40 (±2.66) | 14.33 (±2.67) | 14.57 (±2.43) | 15.17 (±2.89) | 14.93 (±2.58) | 15.33 (±2.73) | 14.90 (±2.62) |
| Matrix Metalloproteinase-9 (MMP-9; ng/ml) | 32.00 (±1.15) | 33.67 (±1.67) | 120.33 (±7.97) | 171.67 (±12.3) | 168.00 (±15.0) | 170.67 (±13.4) | 169.00 (±11.2) | 159.00 (±14.2) |
| Monocyte Chemotactic Protein 1 (MCP-1; pg/ml) | 303.33 (±70.3) | 301.33 (±49.2) | 341.67 (±72.2) | 431.67 (±100.0) | 441.67 (±92.6) | 435.33 (±104) | 421.33 (±91.8) | 440.67 (±84.0) |
| Myeloperoxidase (MPO; ng/ml) | 272.00 (±47.6) | 253.67 (±26.6) | 1673.33 (±367) | 5046.67 (±200) | 5500.00 (±297) | 5483.33 (±284) | 5060.00  (±204) | 3803.33 (±799) |
| Myoglobin (ng/ml) | 36.33 (±7.75) | 34.67 (±6.84) | 37.67 (±8.35) | 37.67 (±7.88) | 38.67 (±8.88) | 35.00 (±8.02) | 35.33 (±7.69) | 36.33 (±7.69) |
| Neuron-Specific Enolase (NSE; ng/ml) | 0.96 (±0.37) | 0.82 (±0.29) | 1.53 (±0.34) | 1.59 (±0.39) | 1.53 (±0.49) | 1.47 (±0.37) | 1.49 (±0.37) | 1.60 (±0.35) |
| Plasminogen Activator Inhibitor 1 (PAI-1; ng/ml) | 47.67 (±5.49) | 45.33 (±7.80) | 96.00 (±11.7) | 142.00 (±18.2) | 145.00 (±14.2) | 132.33 (±16.4) | 128.67 (±8.29) | 105.00 (±11.2) |
| Prostate-Specific Antigen, Free (PSA-f; ng/ml) | 0.21 (±0.12) | 0.21 (±0.11) | 0.24 (±0.13) | 0.26 (±0.14) | 0.27 (±0.15) | 0.28 (±0.16) | 0.29 (±0.15) | 0.27 (±0.14) |
| Pulmonary and Activation-Regulated Chemokine (PARC; ng/ml) | 70.33 (±10.3) | 67.33 (±8.35) | 74.33 (±11.4) | 74.00 (±12.0) | 74.33 (±11.9) | 68.67 (±12.2) | 69.67 (±11.7) | 68.67 (±12.3) |
| Serotransferrin (Transferrin; mg/dl) | 187.00 (±11.1) | 190.00 (±9.85) | 193.00 (±11.0) | 197.00 (±9.17) | 192.33 (±6.06) | 181.33 (±5.61) | 184.67 (±7.06) | 196.33 (±12.4) |
| Serum Amyloid P-Component (SAP; μg/ml) | 11.23 (±2.28) | 11.70 (±2.81) | 11.73 (±2.78) | 11.43 (±2.63) | 12.17 (±2.89) | 11.90 (±2.74) | 11.30 (±2.76) | 11.77 (±2.87) |
| Sex Hormone-Binding Globulin (SHBG; nmol/L) | 43.00 (±10.3) | 46.33 (±10.1) | 43.33 (±11.3) | 47.33 (±10.4) | 51.00 (±11.8) | 43.00 (±11.9) | 44.67 (±9.53) | 47.67 (±12.2) |
| Stem Cell Factor (SCF; pg/ml) | 486.33 (±20.3) | 517.00 (±10.0) | 455.67 (±20.7) | 435.00 (±17.9) | 435.00 (±17.9) | 486.33 (±27.1) | 445.33 (±10.3) | 455.33 (±36.9) |
| T-Cell-Specific Protein RANTES (RANTES; ng/ml) | 6.63 (±2.28) | 5.93 (±2.00) | 10.13 (±1.44) | 12.97 (±2.68) | 13.30 (±2.22) | 12.37 (±2.70) | 12.27 (±1.83) | 10.53 (±1.28) |
| Thrombospondin-1 (ng/ml) | 10050.00 (±2704) | 10566.67 (±2340) | 25566.67 (±4627) | 30966.67 (±3465) | 30433.33 (±4404) | 31966.67 (±4894) | 37166.67 (±4479) | 30933.33 (±4983) |
| Thyroid-Stimulating Hormone (TSH; μIU/ml) | 0.94 (±0.10) | 0.97 (±0.14) | 0.92 (±0.13) | 0.91 (±0.12) | 0.94 (±0.12) | 0.99 (±0.11) | 0.96 (±0.14) | 0.93 (±0.17) |
| Thyroxine-Binding Globulin (TBG; μg/ml) | 33.33 (±0.67) | 34.33 (±1.33) | 34.00 (±1.00) | 33.67 (±1.45) | 35.00 (±2.08) | 33.00 (±2.08) | 30.67 (±1.33) | 34.33 (±2.91) |
| Tissue Inhibitor of Metalloproteinases 1 (TIMP-1; ng/ml) | 73.33 (±4.41) | 72.33 (±4.06) | 89.33 (±7.84) | 108.33 (±5.36) | 104.00 (±4.93) | 95.33 (±5.33) | 95.67 (±4.70) | 90.67 (±7.13) |
| Transthyretin (TTR; mg/dl) | 24.00 (±1.00) | 23.00 (±1.73) | 25.33 (±2.33) | 25.33 (±1.67) | 24.33 (±1.20) | 22.33 (±1.67) | 24.00 (±2.08) | 25.67 (±1.20) |
| Tumor Necrosis Factor alpha (TNF-alpha; pg/ml) | <14* | <14* | 32.00 (±17.4) | 1623.00 (±395) | 1880.00 (±174) | 1489.67 (±358) | 1763.33 (±106) | 1670.00 (±81.4) |
| Tumor Necrosis Factor beta (TNF-beta; pg/ml) | <45* | <45* | <45* | <45* | <45* | <45* | <45* | <45* |
| Tumor necrosis factor receptor 2 (TNFR2; ng/ml) | 5.07 (±0.07) | 4.83 (±0.09) | 6.20 (±0.20) | 7.17 (±0.14) | 7.40 (±0.21) | 6.67 (±0.07) | 6.80 (±0.11) | 6.93 (±0.13) |
| Vascular Cell Adhesion Molecule-1 (VCAM-1; ng/ml) | 413.67 (±24.0) | 396.33 (±7.42) | 418.00 (±29.8) | 417.67 (±15.8) | 426.00 (±22.0) | 398.00 (±26.4) | 411.33 (±20.1) | 397.33 (±24.3) |
| Vascular Endothelial Growth Factor (VEGF; pg/ml) | 84.00 (±8.08) | 85.33 (±6.96) | 151.00 (±22.3) | 300.67 (±46.0) | 305.67 (±44.9) | 278.33 (±39.1) | 290.33 (±39.8) | 254.33 (±36.8) |
| Vitamin D-Binding Protein (VDBP; μg/ml) | 241.33 (±75.1) | 237.00 (±78.7) | 256.67 (±85.1) | 246.00 (±75.0) | 244.33 (±81.4) | 227.33 (±78.4) | 221.00 (±73.2) | 235.67 (±73.9) |
| von Willebrand Factor (vWF; μg/ml) | 47.33 (±20.6) | 49.67 (±21.5) | 59.67 (±27.8) | 53.67 (±21.9) | 56.00 (±24.8) | 50.33 (±20.9) | 54.67 (±19.2) | 65.33 (±28.1) |

*S. aureus* was cultured in human heparinized blood up to 2 h. Accumulation of proinflammatory molecules in plasma was evaluated by quantitative, multiplexed immunoassays (HumanMAP v2.0; Myriad RBM) as described in Materials and Method. Data represents average of 3 donors ±SEM.

* value is below Lower Limit of Quantitation (LLOQ)-lowest concentration of an analyte in a sample that can be reliably detected and at which the total error meets the laboratory’s requirements for accuracy

**^#^** only 1 donor out of 3 had values above LLOQ
